# Supplementary material for: Curated and harmonised transcriptomics datasets of interstitial lung diseases
Source: Data Brief. 2025 Oct 14;63:112139. doi: 10.1016/j.dib.2025.112139 (PMC12581653; doi:10.1016/j.dib.2025.112139)
Supplement: Supplementary file 1 [file mmc1.zip › Supplementary_material/RNA-seq/fastQC reports/GSE169500/SRR14057104_fastqc.html]

SRR14057104.fastq.gz FastQC Report 

FastQC Report

Mon 5 Feb 2024  
SRR14057104.fastq.gz

## Summary

- Basic Statistics
- Per base sequence quality
- Per sequence quality scores
- Per base sequence content
- Per sequence GC content
- Per base N content
- Sequence Length Distribution
- Sequence Duplication Levels
- Overrepresented sequences
- Adapter Content

## Basic Statistics

| Measure | Value |
| --- | --- |
| Filename | SRR14057104.fastq.gz |
| File type | Conventional base calls |
| Encoding | Sanger / Illumina 1.9 |
| Total Sequences | 20760752 |
| Sequences flagged as poor quality | 0 |
| Sequence length | 25-369 |
| %GC | 51 |

## Per base sequence quality

## Per sequence quality scores

## Per base sequence content

## Per sequence GC content

## Per base N content

## Sequence Length Distribution

## Sequence Duplication Levels

## Overrepresented sequences

| Sequence | Count | Percentage | Possible Source |
| --- | --- | --- | --- |
| ACACTGAGGACTCTGTTCCTCCCCTTTCCGCCTAGGGGAAAGTCCCCGGA | 434094 | 2.0909358196658774 | No Hit |
| GGGAATGTCTACGTGCGTATGCACGTGGCACTCTCTGCCCGAGGTCCGGG | 376794 | 1.8149342567167124 | No Hit |
| GGGCCACGAGCTGAGTGCGTCCTGTCACTCCACTCCCATGTCCCTTGGGA | 263969 | 1.2714809174542425 | No Hit |
| AAGCTCAGGGAGAGCCCTGTTAGGGCCGCCTCTGGCCCTAGTCTCAGACC | 235502 | 1.1343616069398643 | No Hit |
| GCTGCTACAGGAGAATAGCAGACAGGTATAGTTAAGAAACAACTTTATTT | 138688 | 0.6680297515234516 | No Hit |
| CTAAGTGCTGTGTTGTCGTTCCCCCTGCTTAAAATAAAGTTGTTTCTTAA | 132231 | 0.636927795293735 | No Hit |
| TTTACACCTGCTAGGGTGTTCAAAGGTCAGTGCTATAGAAATTCAGTATC | 117255 | 0.5647916799930947 | No Hit |
| TCTGTCTTCTTCAGTTTCGACTTATCGAATTTCTCGATCTCAGCCATATC | 100477 | 0.48397572496410535 | No Hit |
| AAAGCCAAGAAAACCAACGATGCCAGATACTGAATTTCTATAGCACTGAC | 78203 | 0.376686740441772 | No Hit |
| TCTTTCTGGCCTGGAGGCTATCCAGCGTACTCCAAAGATTCAGGTTTACT | 65347 | 0.31476220129213045 | No Hit |
| TACTCGTGCGCCTCGCTTTGCTTTTCCTCCGCAACCATGTCTGACAAACC | 63872 | 0.30765744901726105 | No Hit |
| TCTGCAATGCAATCACAATGCCCAAACTAGACCTGCCATTTCTCACACAC | 62173 | 0.2994737377528521 | No Hit |
| AAGACCAACGTCAAGGCCGCCTGGGGTAAGGTCGGCGCGCACGCTGGCGA | 60058 | 0.28928624550786985 | No Hit |
| ACATAGCAATTCAGGAAATTTGACTTTCCATTCTCTGCTGGATGACGTGA | 54060 | 0.2603951918504686 | No Hit |
| TTGGTGGTGGGGAAGGACAGGAACATCCTCTCCAGGGCCTCCGCACCATA | 46599 | 0.22445718729263756 | No Hit |
| TTTTTGTTGCCTGTAAAGTGCTCTGGCATTGCCTGAGGATAGATGAGAAA | 45441 | 0.21887935465921465 | No Hit |
| TGTTGTGCTAAACGCTGGGAGGGTACAAGTTGGTCATTCCTAAATCTGTG | 44897 | 0.21625902568461874 | No Hit |
| ATTCTTCCTACCCATGAGCATGGAATGTTCTTCCATTTGTTTGTATCCTC | 43577 | 0.20990087449626102 | No Hit |
| AGCGGTTCCGCTGCCCTGAGGCACTCTTCCAGCCTTCCTTCCTGGGCATG | 40601 | 0.19556613363523634 | No Hit |
| CCACCTTCTCCATCGGCTCCACTGGCCTCGTGGTGTATGACTACCAGCAG | 39612 | 0.19080233702517135 | No Hit |
| ACACTGAGGACTCTGTTCCTCCCCTTTCCGCCTAGGGAAAGTCCCCGGAC | 39318 | 0.18938620335140077 | No Hit |
| TCACACTTCATGATGGAGTTGAAGGTAGTTTCGTGGATGCCACAGGACTC | 38459 | 0.18524858829776494 | No Hit |
| TGGCTCTCCCAGAGGCAGAAGGACAATGGCTGTTTCAGGAGCTCTGGGTC | 38008 | 0.18307621997507606 | No Hit |
| ATGATGTAGCAGCAGGTGCCAGGGGCTGGCTTGTAGGCGATCAGCAGCTG | 36053 | 0.173659412722622 | No Hit |
| TGTTCCCTCTCCTCATGAGATTGGTGAAGAAAGTATTTGGCAAAGTTCTT | 35374 | 0.1703888182855804 | No Hit |
| AAAACAGCGTCTTACTGGGGAGGGATATGTGCTTTCTCATCTATCCTCAG | 34451 | 0.16594292923493328 | No Hit |
| TGTCCATGTCTTACTACTTTGACCGCGATGATGTGGCTTTGAAGAACTTT | 32497 | 0.15653093876368254 | No Hit |
| GGTTATAATCATTGGCAATGTTGTAATCAACAACCCATGTTATTTCTACA | 32033 | 0.15429595228535076 | No Hit |
| ACACCCACCGCAACTGTCTGTCTCATATCACGAACAGCAAAGCGACCCAA | 31335 | 0.15093383900544644 | No Hit |
| TGGTTCCTGGCAAGCCCATGTGTGTTGAGAGCTTCTCAGACTATCCACCT | 30242 | 0.1456690971502381 | No Hit |
| GAGAGGGTCACTTCATCTTCTACTCCTCCCTTTATGGCATTGTTGAGCAG | 30082 | 0.1448984121577099 | No Hit |
| TGAATCTTGAACTGAGTTCCACTTGTAAACTTCTTGTTTCTTGTGGTTCC | 27238 | 0.13119948641552098 | No Hit |
| CTTCCGCATAGCTGCTGTGGTCAAAAAGGAGCCCAGAGTGACAGTTTTCC | 26337 | 0.1268595665513465 | No Hit |
| TCAGAAAGGTCACGTCCTGCTCTTGGTCCTTGTCAGTGCCATGTTCTGTG | 25090 | 0.12085304039082977 | No Hit |
| TGACTGGTGATGCTGATATTGGAGTGCATCTGAGGCTGTTGTAGAAATAA | 24372 | 0.11739459148685943 | No Hit |
| TAGGACACTTCTGCCAAAGGAACTCGTGCACAGCACCACAGAACATGGCA | 23683 | 0.11407582923778485 | No Hit |
| ACACTGAGGACTCTGTTCCTCCCCTTCCGCCTAGGGGAAAGTCCCCGGAC | 23387 | 0.11265006200160765 | No Hit |
| GGCCACGAGCTGAGTGCGTCCTGTCACTCCACTCCCATGTCCCTTGGGAA | 22680 | 0.10924459769087362 | No Hit |
| TCTTCAAGGAGAACTACAAACCACTGCTCAAGGAAATAAAAGAGGATACA | 22645 | 0.10907601034875808 | No Hit |
| AAACCAGACATGGGGGAAATCGCCAGCTTCGATAAGGCCAAGCTGAAGAA | 22089 | 0.10639787999972256 | No Hit |
| AGTGTGGCGATTCCTCAGGGATCTAGAACTAGAAATACCATTGACCCAGC | 21975 | 0.1058487669425462 | No Hit |
| CACTTGGCTTCACCCGTGTGACTTTCGTAACGGGGAGAGAGAGAAAAGAT | 21798 | 0.10499619666956188 | No Hit |
| AGAGGCCACAGTAAGGCCCATCCGAGGTCCTGAGGAGATCTTTTCTCTCT | 21516 | 0.1036378643702309 | No Hit |
| GAAGACGTACCTTTGTTGGAAGACATACCTTTGTTGGAAGACGTACCTTT | 21069 | 0.10148476317235523 | No Hit |
| AATACCAAGACCTGCTCAATGTTAAGATGGCCCTTGACATTGAGATTGCC | 21062 | 0.1014510457039321 | No Hit |
| CAGCATGATTTATAGTCCTTTGGGTATATACCCAGTAATGGGATGGCTGG | 20779 | 0.10008789662339784 | No Hit |

## Adapter Content

Produced by FastQC (version 0.11.7)
